# Can we use meat inspection data for animal health and welfare surveillance?

**DOI:** 10.3389/fvets.2023.1129891

**Published:** 2023-05-10

**Authors:** Arianna Comin, Anita Jonasson, Ulrika Rockström, Arja Helena Kautto, Linda Keeling, Ann-Kristin Nyman, Ann Lindberg, Jenny Frössling

**Affiliations:** ^1^Department of Epidemiology and Disease Control, Swedish National Veterinary Institute, Uppsala, Sweden; ^2^Farm and Animal Health, Uppsala, Sweden; ^3^Department of Biomedicine and Veterinary Public Health, Swedish University of Agricultural Sciences, Uppsala, Sweden; ^4^Department of Animal Environment and Health, Swedish University of Agricultural Sciences, Uppsala, Sweden; ^5^Växa, Department of Research and Development, Stockholm, Sweden; ^6^Department of Animal Environment and Health, Swedish University of Agricultural Sciences, Skara, Sweden

**Keywords:** meat inspection, beef cattle, finishing pigs, lesions, variance partitioning analysis, animal health surveillance

## Abstract

Ante- and post-mortem inspections at abattoir were originally introduced to provide assurance that animal carcasses were fit for human consumption. However, findings at meat inspection can also represent a valuable source of information for animal health and welfare surveillance. Yet, before making secondary use of meat inspection data, it is important to assess that the same post-mortem findings get registered in a consistent way among official meat inspectors across abattoirs, so that the results are as much independent as possible from the abattoir where the inspection is performed. The most frequent findings at official meat inspections of pigs and beef cattle in Sweden were evaluated by means of variance partitioning to quantify the amount of variation in the probabilities of these findings due to abattoir and farm levels. Seven years of data (2012–2018) from 19 abattoirs were included in the study. The results showed that there was a very low variation between abattoirs for presence of liver parasites and abscesses, moderately low variation for pneumonia and greatest variation for injuries and nonspecific findings (e.g., *other lesions*). This general pattern of variation was similar for both species and implies that some post-mortem findings are consistently detected and so are a valuable source of epidemiological information for surveillance purposes. However, for those findings associated with higher variation, calibration and training activities of meat inspection staff are necessary to enable correct conclusions about the occurrence of pathological findings and for producers to experience an equivalent likelihood of deduction in payment (independent of abattoir).

## 1. Introduction

In Europe, all food-producing animals are subjected to official a*nte-* and *post-mortem* inspections at slaughter (1). Such activities were originally introduced to provide assurance that animal carcasses were fit for human consumption (2). However, it was subsequently recognized that such inspections also play an integral role in assessment of animal health and zoo-sanitary status, as well as in detection of certain welfare conditions (3), as they often reflect the standard of the housing and husbandry of the animal during the production period (4). Nevertheless, the information collected at meat inspection is not yet exploited to its full potential (5).

An extensive review of current practices of meat inspection in the European Union (EU) (3) reports that traditional meat inspection according to EU regulations is in principle conducted in most Member States. However, the regulation is not necessarily fulfilled with respect to all detailed requirements and inadequate ante- and post-mortem inspection still occurs. In addition, the EU legislation concerning judgment criteria at meat inspection is mostly generic, allowing flexibility on one side but also subjectivity on the other side (6). Such variability in meat inspection procedures leaves an open question about the suitability of data collected during meat inspection for animal health and welfare surveillance. In particular, it is not yet clear which inspection findings should be included in a surveillance system at the abattoir and whether data collected at meat inspection are usable for the purpose. For the information to be valuable, it is imperative that the post-mortem findings are accurately diagnosed and consistently detected, so that the result of the inspection does not strongly depend neither on the individual inspector nor the abattoir where the inspection takes place.

The accuracy of routine meat inspection of pigs has been evaluated in previous studies. Bonde et al. (7) estimated the sensitivity and specificity of post-mortem inspection performed by meat inspectors in comparison to veterinary researchers. Schleicher et al. (8) estimated the amount of variation in the recording of post-mortem findings that can be attributed to the individual official meat inspectors. In both cases, the evaluations were done at inspector level and involved one single abattoir, making it difficult to generalise the results to the common meat inspection practice. Van Staaveren et al. (9) investigated the use of carcass lesions as indicators of animal health and welfare on farm. However, the inspection at abattoir was performed by one of the authors using a pre-defined scoring system, which might not reflect the practice of meat inspection under working conditions. More recently, Klinger et al. (10) assessed the impact of farm of origin, abattoir and time of the year on the prevalence of post-mortem findings recorded in 66 Austrian abattoirs. The pathological findings were grouped into five main categories (i.e., respiratory system and heart, abdominal organs, skin and locomotor system, other pathologies, and slaughter-related lesions) which does not allow to assess the accuracy of individual lesions for their potential use in surveillance. When it comes to meat inspection in cattle, the body of literature is slimmer. Veldhuis et al. (11) quantified the associations between meat inspection findings and farm of origin characteristics in dairy cattle slaughtered in one abattoir, concluding that seven indicators provided added value to existing cattle health surveillance components, however the implementation will be challenging due to lack of standardization between abattoirs. Denwood et al. (12) estimated the farm and abattoir variation in meat inspection of beef cattle, concluding that the sensitivity of meat inspection is affected by differences in the working practices between abattoirs, resulting in biased prevalence estimates.

The aim of this study was to evaluate the quality of meat inspection data collected from several abattoirs under real working conditions, to assess which findings are currently consistently detected among abattoirs and might therefore become suitable surveillance information. To reach this objective, we estimated the amount of variation in the registered post-mortem findings of pigs and cattle that can be attributed to the abattoir where the carcasses are inspected.

## 2. Materials and methods

We used 7 years of data on all findings recorded in beef cattle and finishing pigs slaughtered from 2012 to 2018 in the 19 largest abattoirs in Sweden, slaughtering altogether approximately 80% of the animals in the country. Eight abattoirs slaughtered only cattle, five only pigs and six both cattle and pigs. Most farms slaughtered their animals in a single abattoir, while one-fifth supplied two abattoirs and only a few supplied three or more. More information about the number of animals slaughtered, individual batches received, and different farms served by each abattoir as well as the distribution of farms by number of abattoirs used is given in the Supplementary material (Supplementary Tables S1, S2). To reduce the variation due to animal characteristics we restricted the beef cattle population to a subcategory named *young bulls*, which identifies non-castrated male beef cattle aged 24 months. Finishing pigs included females that have never been pregnant and castrated males, aged 5–6 months and weighing around 120 kg at slaughter.

In Sweden, meat inspection is performed by official veterinarians and auxiliaries employed by the Swedish Food Agency. Official inspectors undergo specific training with regular follow ups and perform meat inspection according to EU Regulations, adopting a common national frame for condemnation (13). Meat inspectors observe carcass parts on the slaughter line before dressing and weighing. The time allowed for postmortem inspection depends on multiple factors, including the number of inspectors involved. For instance, in the case of pigs, if the speed of the slaughter line is 450 pigs per hour, four meat inspectors are present for visual control. However, if the speed is 90 pigs per hour, only one meat inspector conducts visual inspection. In situations where the speed is between 86 and 100 pigs per hour, there is a mere 36–42 s per carcass and organs available for inspection. The speed of the line, and therefore the number of inspectors, can vary significantly between abattoirs. Unfortunately, we were unable to obtain such information. At meat inspection, up to five different findings can be recorded at carcass level using a standardized coding system including 37 different lesions or abnormalities. If more than five lesions are observed, not all of them will be recorded. The full list of lesions/abnormalities and their description is provided in the Supplementary material (Supplementary Table S3).

Data were aggregated at batch level, which was defined as a group of animals delivered from an individual farm to a given abattoir on a given date. In total, 113,305 cattle and 166,658 pig batches were investigated. The mean batch size (±standard deviation) was 7.5 ± 7.5 for young bulls (min = 1, max = 121, median = 5) and 100 ± 87 for finishing pigs (min = 1, max = 758, median = 75).

Only lesions whose overall prevalence at carcass level in the study period exceeded 0.5% were included in the analysis. Their description, as provided to meat inspectors for their assessment, is given in Table 1.

**Table 1:** Description of the meat inspection findings investigated in this study, as provided to meat inspectors for their assessment.

Location	Diagnosis	Description
Carcass	Joint injury	Includes all types of joint injuries, both infectious and non-infectious. Acute septic arteritis is registered as “Sepsis”
	Chronic injury	Older musculoskeletal traumatic injury, for example wounds, bruises and fractures
	Acute injury	Recent musculoskeletal traumatic injury, for example wounds, bruises and fractures
	Tail lesion, pigs only	Obvious bite marks on the tail and other tail injuries where at least 50% of the tail lost. Also applies to fully healed injuries regardless of the cause
	Abscess (other than in liver)	Macroscopic finding in the carcass or organs. Liver abscesses must be registered separately
Lungs	Mycoplasma-like lesions	In swine, mycoplasma-like lesions with a minimum presence of moderate pneumonia in at least three lung lobes or high-grade pneumonia in one lobe The code is reserved for pigs only. Similar pneumonias in other animal species must be registered as “Other pneumonia”
	Fibrinous pneumonia	Pneumonia with typical changes and location suggesting the presence of *Actinobacillus pleuropneumoniae* in pigs
	Other pneumonia	The etiology of pneumonias in cattle and sheep is difficult to assess macroscopically and therefore is must be registered as “Other pneumonia.” The code must also be used to register pyemic pneumonia in pigs, e.g., in connection with tail lesions
	Pleurisy/pericarditis	Presence of acute pleuritis and focal chronic adhesions when the fibrous scar(s) on chest wall are ≥3 cm. Or presence of acute, exudative pericarditis
Liver	Common liver fluke (*Fasciola hepatica*)	Presence of grey-brown, flat, flounder-shaped parasites sized 2–10 mm (juvenile) to 20–30 mm (adults)
	Lancet liver fluke (*Dicrocoelium dendriticum*)	Presence of semi-transparent, oblong (lanceolate) parasites sized 5–15 mm
	Parasitic liver damage	Refers to so-called “white spots” in pigs and parasitic granulomas in other slaughter animals as well as bile duct changes and other changes secondary to parasitic infection (without presence of parasites). Parasitic granulomas are nodular, solid nodules containing large amounts of eosinophilic granulocytes, which give the nodules a greenish color. The nodules may become tubercle-like through cheesiness and calcification
	Liver abscess	Presence of abscesses in the liver
	Pleurisy and perihepatitis	In case of pleuritic spread as described for “Pleurisy/pericarditis” and at the same time perihepatitis which causes unfitness of the whole the liver (local condemnation). Presence of perihepatitis only is recorded as “Other liver damage”
	Other liver damage	When the liver is condemned due to findings not included in any other existing code. For example: telangiectasia, perihepatitis, cirrhosis, stasis. Perihepatitis is registered as “Pleurisy and perihepatitis “when pleurisy is present at the same time. Fatty liver is registered with its own code (“Fatty liver”)
Any	Other cause	In the event of pathological changes that do not have their own code and that need additional post-mortem inspection procedures by an official veterinarian for final decision (total/local condemnation)

The full list of findings is available in the Supplementary material (Supplementary Table S3).

In order to quantify the proportion of total variance in the outcome (i.e., presence of a given post-mortem finding in a slaughter batch) attributable to one or more effects, we fitted a logistic mixed model with cross-classified random effects for each of the lesions as proposed by Denwood et al. (12), for pigs and cattle separately. The response variable *Y_i_* (i.e., the number of a given post-mortem finding for batch *i*) was described using a Binomial distribution, according to the fitted probability p*_i_* and total number of recordings *N_i_* in the batch *i*. The model was fitted under the assumption that the probability of a specific post-mortem finding in a slaughter batch (*p_i_*) may depend on farm characteristics and the abattoir where the meat inspection is carried out, resulting in observations being clustered between farms and/or abattoir. Therefore, the intercept and two cross-classified random effects, one for the farm and one for the abattoir, were included. Given that the post-mortem finding occurrence showed a zero-inflated distribution, we fitted an additional random effect at batch level, to correct for overdispersion as suggested by Browne et al. (14) and Harrison (15). The general form of the model is as follows:


$$Y_{i}\sim \text{Binomial}\left(p_{i},N_{i}\right)$$

$$\text{Logit}\left(p_{i}\right)=Z+A_{k}+F_{j}+B_{i}$$

Where, *p_i_* is the probability of observing the outcome in batch *i*, *Z* the common intercept, *A* the random effect of abattoir *k*, F the random effect of farm *j* and *B* the random effect of batch *i*.

All models were fitted using the *glmer* function of the “lme4” package (16) in *R* (17).

We then calculated the variance partitioning coefficients (VPCs) that quantify the extent of clustering in the meat inspection data, or in other words, the proportion of total variance in the outcome that is attributable to the random effects. The percentage of variation explained by the abattoir is given by:


$${\text{VPC}}_{A}=\frac{{\text{Var}}_{A}}{{\text{Var}}_{A}+{\text{Var}}_{F}+{\text{Var}}_{B}+{\text{Var}}_{\varepsilon }}\times 100$$

while the percentage of variation explained by the farm is given by:


$${\text{VPC}}_{F}=\frac{{\text{Var}}_{F}}{{\text{Var}}_{A}+{\text{Var}}_{F}+{\text{Var}}_{B}+{\text{Var}}_{\varepsilon }}\times 100$$

where, ${\text{Var}}_{A}$ and ${\text{Var}}_{F}$ are the variance of random effects at abattoir and farm level estimated from the model, ${\text{Var}}_{B}\;$ the overdispersion parameter (i.e., variance at batch level) and ${\text{Var}}_{\varepsilon }$ the residual variance. The latter was approximated using the latent variable method (18) assuming that every carcass has a certain *propensity* to have a given post-mortem finding, but only those whose propensity exceeds a certain threshold actually get it. This approach well suits the meat inspection process, where a particular finding is recorded only when its size/severity is big enough to note it. The unobserved (latent) individual variable follows a logistic distribution with individual level variance equal to $\pi^{2}/3$, which is independent of the value of any possible linear predictor (14, 19).

Ideally, there should be a minimal variation in the way carcasses are assessed among abattoirs, meaning that ${\text{VPC}}_{A}$ should be close to zero. Besides the actual estimate of ${\text{VPC}}_{A}$, it is important to assess its relative magnitude compared to ${\text{VPC}}_{F}$. This relation is expressed by the ${\text{VPC}}_{A:F}$ ratio, which shows how much bigger/smaller the variation at abattoir level is compared to the variation at farm level.

## 3. Results

Overall, the most frequent findings at meat inspection involved lungs and liver. They were pneumonia (5.5%), pleurisy/pericarditis (5.1%) and common liver fluke (4.3%) for young bulls (Table 2) and pleurisy/pericarditis (13.6%), parasitic liver damage (4.9%) and Mycoplasma-like lesions (3.3%) for finishing pigs (Table 3). Abscesses and injuries were also reported, albeit less frequently. In general, the frequency of occurrence of abnormal findings in cattle and pig carcasses in Sweden was quite low, with only 10 lesions (in cattle) and 13 lesions (in pigs) among the 37 monitored ones showing a prevalence higher than 0.5% in the 7-year period.

**Table 2:** Variance partitioning for most frequent lesions recorded in 2012–2018 at meat inspection of young bulls, sorted by frequency of occurrence (*N* = 113,305 slaughter batches).

Lesion	Prevalence at individual carcass level (%)	VPC_A_ (%)	VPC_F_ (%)	VPC_A:F_ (ratio)
Other pneumonia	5.46	4.67	12.84	0.36
Pleurisy/pericarditis	5.06	8.21	8.39	0.98
Common liver fluke	4.30	0.04	71.63	0
Chronic injury	3.40	16.32	9.13	1.79
Other liver damage	3.20	8.59	3.23	2.66
Liver abscesses	3.03	0.01	16.81	0
Parasitic liver damage	2.55	15.19	9.58	1.59
Lancet liver fluke	1.60	0.02	37.45	0
Joint injury	0.71	10.65	10.10	1.06
Abscess	0.68	0	0	–
Traumatic peritonitis	0.64	3.78	6.15	0.61

**Table 3:** Variance partitioning for most frequent lesions recorded in 2012–2018 at meat inspection of finishing pigs, sorted by frequency of occurrence (*N* = 166,658 slaughter batches).

Lesion	Prevalence at individual carcass level (%)	VPC_A_ (%)	VPC_F_ (%)	VPC_A:F_ (ratio)
Pleurisy/pericarditis	13.58	0.95	19.49	0.05
Parasitic liver damage	4.92	1.34	31.03	0.04
Mycoplasma-like lesions	3.28	6.39	17.74	0.36
Tail damage	2.71	3.97	7.61	0.52
Abscess (other than liver)	1.39	1.75	2.21	0.79
Other cause	1.10	20.48	8.37	2.45
Other pneumonia	1.04	4.95	5.05	0.98
Pleurisy and perihepatitis	0.86	2.24	8.37	0.27
Other liver damage	0.82	3.33	7.3	0.46
Joint injury	0.74	6.17	7.31	0.84
Chronic injury	0.72	8.40	3.26	2.58
Fibrinous pneumonia	0.69	0	0	–
Acute injury	0.58	17.93	1.62	11.06

### 3.1. Young bulls

The results of variance partitioning for young bulls are reported in Table 2. Abscesses (other than liver) and liver parasites showed nearly no variation among abattoirs (VPC_A_ ≈ 0), meaning that they were consistently detected across the investigated abattoirs. It is interesting to notice that liver flukes were consistently detected among abattoirs despite the regional differences in the prevalence of *Fasciola hepatica* and *Dicrocoelium dendriticum*; differences that were captured by a high variation between farms (VPC_F_ = 37–71%).

Traumatic peritonitis and pneumonia showed a moderately low variation at abattoir level (VPC_A_ < 5%), which was nonetheless lower than the variation at farm level (VPC_A:F_ < 1). Injuries and liver damages were the lesions with the highest VPC_A_ (8–15%), which was also higher than VPC_F_, meaning that the probability of a young bull being identified with such lesions was strongly influenced by the abattoir where the inspection took place.

### 3.2. Finishing pigs

The results of variance partitioning in finishing pigs are reported in Table 3. Fibrinous pneumonia and pleurisy/pericarditis showed the lowest variation among abattoirs (VPC_A_ < 1%). Abscesses, liver damages, pneumonias and tail damages showed a moderately low variation at abattoir level (VPC_A_ < 5%), which was nonetheless lower than the variation at farm level (VPC_A:F_ < 1). Injuries and unspecific lesions (i.e., “other cause”) were associated with the highest VPC_A_ (8–20%), which was also higher than VPC_F._

## 4. Discussion

The probability of observing a given post-mortem finding in a slaughter batch depends on several factors that have an impact at different stages of the supply chain, such as the animal level, farm level, and/or abattoir level. Knowing the sources of variation of the post-mortem findings might help assessing the quality of meat inspection by separating individual- and farm-related factors from the accuracy of the detecting procedure (i.e., abattoir-related factors, including both detection ability of the inspectors and slaughter line conditions).

In our study, abscesses, liver parasites and pneumonia seemed to be consistently diagnosed, while injuries, liver damages and nonspecific findings showed a greater variation among abattoirs. It seems therefore reasonable to infer that some post-mortem findings are easier to detect and classify while others are more prone to subjective interpretation. In the latter case, the level of training and experience of the meat inspector plays a strong role in the correct identification of the findings, in conjunction with abattoir-specific slaughter line configurations (e.g., speed, accessibility of carcasses and offal for meat inspectors, luminosity of meat inspection platform, etc.). However, sometimes it is just difficult to correctly assess a specific finding. If we take injuries as an example, the distinction between acute and chronic lesions is not always clear-cut and it might require histopathological diagnostics to correctly differentiate between them. In addition, there is currently no clear description of how extensive the injury must be in order to be registered, leading to more subjective evaluations (Table 1).

Whenever a post-mortem finding is difficult to assess—either because the slaughter line conditions do not allow it, the description of the lesion is not clear enough or the finding is intrinsically difficult to assess—the probability of detection depends on the person carrying out the examination, and these findings cannot be directly translated into surveillance data.

According to Kahneman et al. (20), the sources of failure in professional judgments rely on two main components: one is the systematic error (i.e., the average error in judgments) and the other is the noise (i.e., the variability of error in judgements). The former happens across raters (e.g., because the description of a finding is not clear enough and leave room for interpretation) while the latter is more individual-dependent and arises because of personal factors such as cognitive biases, mood, group dynamics and emotional reactions. The systematic error observed in certain post-mortem findings can be reduced through specific training and calibration activities for official meat inspectors. On the other hand, reducing the noise in meat inspection might not be so straightforward, as it involves the personal reactions of each inspector. It is however reassuring to notice that, especially for pigs, the most frequent findings at meat inspection were also those most consistently detected.

Interestingly, the probability of observing abscesses (other than liver) in cattle and fibrinous pneumonia in pigs seemed to be independent from the farm of origin or the abattoir assessing the carcass. For these lesions, in fact, most of the variation (80% and 70%, respectively) was due to the batch itself (see Supplementary Tables S4, S5), suggesting that factors such as individual animal variation, failed vaccination, existing infections, stress due to transport and/or seasonality were playing a major role.

Assessment of cattle and pig lesions showed both similar and contrasting trends. In both cases, abscesses (other than liver) were consistently detected, and injuries were more subjectively evaluated. On the contrary, pleurisy/pericarditis, parasitic liver damage and other liver damage showed a high level of variation among abattoirs in cattle but a high consistency in pigs. This is partly due to different clinical manifestations in the two species (e.g., parasitic liver damage in pigs—aka milk spots—are fairly characteristic and easier to recognize than in cattle) but also to the fact that, beyond the regular training sessions, inspectors working at Swedish pig abattoirs underwent major calibration exercises in 2017 (and subsequently in 2019).

A larger body of literature is available concerning meat inspection in pigs compared to cattle, probably because the pig sector represents one of the most economically important farming sectors in the EU and worldwide (21). Previous studies on the quality of the meat inspection process in pigs reported a moderate-to-high variation both among abattoirs (10, 22) and between official veterinarians carrying out meat inspection (23), confirming that lesions such as abscesses, peritonitis, and milk spots are more consistently detected than others (e.g., skin lesions and hepatitis) (8). In addition, other authors found that pig producers experienced distrust in meat inspection due to inconsistencies among different abattoirs (24) and that a portion of the food business operators in meat, fish or dairy processing considered the food controls non-uniform (25). This highlights the importance of continuous training of meat inspectors as well as regular calibration exercises, especially for those findings that are more prone to subjective evaluation. To this regard, a limitation of this study is that it does not take into account the intra-abattoir variation. Knowing which inspector made the assessment would have enabled us to identify systematic differences among inspectors. In the same way, knowing the slaughter line configuration of each abattoir (e.g., line speed, carcass/offal accessibility, luminosity of control stations, etc.) would have helped interpreting the systematic differences among abattoirs. Unfortunately, none of the above-mentioned information was available. Nevertheless, the abattoir variation can be considered as a proxy for between-inspector variation, given that the inspectors working at a big abattoir tend to be the same. In addition, from a producer point of view, it is important to ensure a fair inspection process irrespective of which abattoir they choose.

Meat inspection data has some intrinsic limitations, not least the fact that it is impossible to identify whether the absence of a given finding is due to lack of occurrence (i.e., true absence), lack of detection (i.e., missed finding) or lack of reporting (e.g., correctly detected by not correctly registered). Nevertheless, like passive surveillance, it can still represent a valuable source of epidemiological information for cattle and pig health and welfare, given that the most frequent findings were also those most consistently reported. However, to enable correct conclusions about disease occurrence and for producers to experience an equivalent likelihood of deduction, calibration and training activities of official meat inspectors are necessary for those findings associated to higher variation within and between abattoirs. For those lesions that are undoubtedly difficult to classify, it could be hypothesized to record a lower level of detail. In case of injuries, for instance, one could just record more generically “injury,” without distinguishing between acute and chronic. However, while less detailed diagnoses may be detected more consistently, they carry less information for surveillance purposes. Within the abovementioned example of injuries, it would be very important to distinguish whether an injury is acute or chronic in the context of animal welfare surveillance, but a generic diagnosis would not allow that. In addition, a generic diagnosis would not allow to identify who is liable for financial deduction for the injury (i.e., the farmer, the transporter or the abattoir, depending on how chronic or acute the injury is). To gain further insight into the issue, we calculated percentage of variation explained by the abattoir for the combination of acute and chronic injury findings in pigs, as they had been recorded as a generic injury. The VPC_A_ for this combined injury was found to be 8.10% (VPC_A:F_ = 3.52), which was lower than the VPC_A_ for the two lesions taken separately. However, it is worth noting that the difference with the VPC_A_ for chronic injuries alone (8.40%) was minor.

Looking for the balance between accuracy and precision of diagnoses at meat inspection, another option could be to support the work of meat inspectors with new digital techniques and artificial intelligence. In particular, artificial intelligence can be used to analyze and process large amounts of data from various sources, such as image processing system, chemical sensors, and microbiological tests to identify patterns and anomalies in meat quality, classify carcasses, detect potential health hazards, and provide real-time feedback to inspectors (26, 27). As a matter of fact, such options are already under development, such for instance the use of artificial intelligence for automatic detection of abattoir lesion (ADAL | F4T Lab) (28), photo artificial intelligence identification of animals (Phaid | F4T Lab ) (29) and scanning animal tattoos on slaughter line for traceability (ReaDop | F4T Lab ) (30).

## Data availability statement

The original contributions presented in the study are included in the article/Supplementary material, further inquiries can be directed to the corresponding author.

## Ethics statement

Ethical review and approval were not required for this study as it did not involve any animals, animal samples nor isolates. The study only made use of data that was previously collected and stored in a private database.

## Author contributions

AC: conceptualisation, methodology and data analysis, writing—original draft, review and editing, and funding acquisition. AJ, UR, AK, LK, A-KN, AL, and JF: conceptualisation, interpretation of results, and review and editing. All authors contributed to the article and approved the submitted version.

## Funding

This work was funded by the Swedish Research Council Formas (grant no. 2017-00593).

## Conflict of interest

The authors declare that the research was conducted in the absence of any commercial or financial relationships that could be construed as a potential conflict of interest.

## Publisher’s note

All claims expressed in this article are solely those of the authors and do not necessarily represent those of their affiliated organizations, or those of the publisher, the editors and the reviewers. Any product that may be evaluated in this article, or claim that may be made by its manufacturer, is not guaranteed or endorsed by the publisher.
