# Supplementary material for: Can we use meat inspection data for animal health and welfare surveillance?
Source: Front Vet Sci. 2023 May 10;10:1129891. doi: 10.3389/fvets.2023.1129891 (PMC10205995; doi:10.3389/fvets.2023.1129891)
Supplement: Supplementary file 1 [file Data_Sheet_1.PDF]

Supplementary Material

Can we use meat inspection data for animal health and welfare surveillance?

Arianna Comin*, Anita Jonasson, Ulrika Rockström, Arja Helena Kautto, Linda Keeling, Ann-Kristin Nyman, Ann Lindberg, Jenny Frössling

*** Correspondence:** Arianna Comin: arianna.comin@sva.se

Supplementary Figures and Tables

Table S1. Number of animals slaughtered, individual batches received, and different farms served by each abattoir during the 7-year period (2012-2018).

	Animals		Batches		Farms	
Abattoir	Young bulls	Finishing pigs	Young bulls	Finishing pigs	Young bulls	Finishing pigs
A01	761 22	2 336 253	11 970	20 661	1 744	414
A02	139 858	2 652 998	17 522	20 663	1 879	368
A03	52 717	504 594	5 423	6 461	338	67
A04	31 663	919 644	5 495	10 888	728	172
A05	39 102	314 154	4 347	4 082	484	102
A06	106 940	1 890 589	14 740	19 101	2 016	306
A07	3 570	-	516	-	117	-
A08	973	-	198	-	108	-
A09	16 8261	-	25 480	-	2 474	-
A10	3 247	-	549	-	188	-
A11	220 347	-	25 287	-	2 638	-
A12	1 045	-	209	-	87	-
A13	5 720	-	1 142	-	413	-
A14	3 435	-	427	-	120	-
A15	-	4 981 557	-	49 010	-	641
A16	-	588 806	-	10 522	-	227
A17	-	783 470	-	9 726	-	269
A18	-	72 322	-	974	-	28
A19	-	1 614 271	-	14 570	-	199
Total	853 000	16 658 658	113 305	166 658	13 334	2 793

Table S2. Distribution of farms by number of abattoirs used during the 7-year period (2012-2018).

Number of different abattoirs utilized by a farm	**Cattle farms**		**Pig farms**	
	number	%	number	%
1	6383	68%	771	53%
2	2139	23%	294	20%
3	643	7%	180	12%
4	163	2%	117	8%
5	32	0.3%	47	3%
6	4	0.04%	25	2%
7	0	0%	10	1%
8	0	0%	1	0.1%
Total	9364	100.00%	1445	100%

Table S3. List and description of codes/diagnoses provided to meat inspectors for their assessment (translated from Swedish). Those marked in bold were included in the study. For the same diagnosis, odd and even codes denote total and partial carcass condemnation, respectively.

Code	***Diagnosis***	***Description***
01/02	Salmonella	Detection of salmonella in organs, lymph nodes or on meat. Confirmatory laboratory diagnosis needed. Secondary contamination included.
03/04	Tuberculosis (*M. bovis and tuberculosis*)	Confirmatory laboratory diagnosis needed. Generalized code 03, local condemnation code 04 (according to Reg. (EU) 2019/627)
05/06	Atypical Mycobacteriosis	Signs of M. avium or suis. Generalized including lungs/spleen total condemnation (05) or primary complex (jaw/jaw lymph nodes and/or liver) local condemnation (06)
09/10	Trichinosis	Confirmatory laboratory diagnosis needed. Always total condemnation in positive cases, code 9.
11/12	Cysticercosis	Detection of one or more fresh or calcified cysts by several incisions in the musculature, referrable to *Cysticercys bovis* (cattle), *C. ovis* (sheep) and *C. cellulosae (pigs)*. Confirmatory laboratory diagnosis needed.
13/14	Echinococcosis	Sampling and confirmatory laboratory diagnosis needed.
15/16	Onchocerchiasis	Subcutaneous, possibly greenish changes in thin skin, flexor tendons and ligaments mainly around the lower joints of the extremities. Similar changes in other locations see code 53/54.
17/18	Erysipelas	Typical skin erythema with rhomboid elevations caused by bacterium *Erysipelothrix rhusiopathiae*
19/20	Sepsis	Sepsis occurs when an infectious lesion involves multiple organ systems/tissues in the form of acute, macroscopically observable miliary/embolic abscesses, petechial-ecchymotic haemorrhages, infarctions and/or miliary necrosis.<br>The term sepsis includes pyemia, viremia, parasitemia, fungemia, and toxemia.
23/24	Antibacterial substances, unauthorized levels of medical products	Refers to antibiotic control as a result of suspected antibiotic treatment. There is then a risk that antibiotics may be present in the meat and pending test results the actual blood batch must be declared unfit for use
25/26	Tumor	Macroscopic finding (mass), lymphoma and possibly leucosis included.
27/28	Traumatic serositis	Includes all pathological changes due to ingestion of sharp foreign body placed in fore stomach, giving changes such as traumatic peritonitis, pericarditis, splenitis, etc.
29/30	**Abscess (other than in liver)**	Macroscopic finding in the carcass or organs. Liver abscesses must be registered separately (85/86)
31/32	**Joint injury**	Includes all types of joint injuries, both infectious and non-infectious. Acute septic arteritis coded as code 19.

33/34	Abnormal appearance	Examples of abnormal appearance are anemia, icterus and insufficient bleeding. If possible, the primary cause must be stated in the decision. However, PSE must be recorded with its own code (35/36)
35/36	PSE, pigs only	Pale color and soft/stringy structure on the cut surfaces of the musculature with presence of liquid discharge (exudate). Subjective assessment. Corresponding changes in cattle (DFD) are not recorded.
37/38	Fatty liver	Hepatic fatty degeneration.
39/40	**Chronic injury**	Older musculoskeletal traumatic injury, for example wounds, bruises and fractures.
41/42	**Acute injury**	Recent musculoskeletal traumatic injury, for example wounds, bruises and fractures.
45/46	Abnormal odor	Examples of abnormal odors are odor of acetone, feed, manure, male sex (boar taint included) and urine. If possible, the primary cause must be stated in the decision.
47/48	Emaciation/leaning	Serous atrophy at coronaria groove area and bone marrow and muscle atrophy (clearly visible parts of skeleton) identified for emaciation (code 47). Cachexia or lean/thin carcasses (not all above recorded) coded as 48.
51/52	**Other cause**	In the event of pathological changes that do not have their own code and that need additional post-mortem inspection procedures by an official veterinarian for final decision (total/local condemnation).
53/54	Parafilariasis	Focal hemorrhages and edema with yellow-green discoloration and metallic odor usually localized to subcutaneous and intramuscular connective tissue on the upper half of the animal from neck to loin.<br>Diagnosis only in cattle (and horse). *Parafilaria* occurs in horses, but is currently only found in Eastern Europe.
57/58	**Tail lesion, pigs only**	Obvious bite marks on the tail and other tail injuries where at least 50 % of the tail lost. Also applies to fully healed injuries regardless of the cause.
61/62	**Swine enzootic pneumonia**	In swine, mycoplasma-like lesions with a minimum presence of moderate pneumonia in at least three lung lobes or high-grade pneumonia in one lobe.<br>The code is reserved for pigs only. Similar pneumonias in other animal species must be registered with code 63/64.
63/64	**Other pneumonia**	The etiology of pneumonias in cattle and sheep is difficult to assess macroscopically. These pneumonias must therefore be registered with code 63/64. The code must also be used to register pyemic pneumonia, e.g. in connection with tail lesions.
71/72	**Fibrinous pneumonia**	Pneumonia with typical changes and location suggesting the presence of *Actinobacillus pleuropneumoniae* in pigs.
73/74	Parasitic pneumonia (lungworm)	Nematodes from a few mm in size to 6-8 cm freely moving in the bronchi and bronchioles and/or red-yellow-gray granulomas in the lung

		parenchyma. In absence of larvae, the finding is registered when at least 10 granulomas are seen or when one granuloma is >1 cm in diameter.
75/76	**Pleurisy/pericarditis**	Presence of acute pleuritis and focal chronic adhesions when the fibrous scar(s) on chest wall are ≥3 cm. Or presence of acute, exudative pericarditis
77/78	**Pleurisy and perihepatitis**	In case of pleuritic spread according to code 75/76 and at the same time perihepatitis which causes unfitness of the whole the liver. Only perihepatitis is recorded with code 87/88 (Other liver damage).
79/80	**Common liver fluke (*Fasciola hepatica*)**	Presence of grey-brown, flat, flounder-shaped parasites sized 2-10 mm (juvenile) to 20-30 mm (adults) 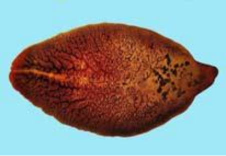
81/82	**Lancet liver fluke (*Dicrocoelium dendriticum*)**	Presence of semi-transparent, oblong (lanceolate) parasites sized 5-15 mm 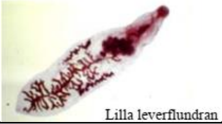
83/84	**Parasitic liver damage**	Refers to so-called "white spots" in pigs and parasitic granulomas in other slaughter animals as well as bile duct changes and other changes secondary to parasitic infection. Parasitic granulomas are nodular, solid nodules containing large amounts of eosinophilic granulocytes, which give the nodules a greenish color. The nodules may become tubercle-like through cheesiness and calcification.
85/86	**Liver abscess**	Presence of abscesses in the liver
87/88	**Other liver damage**	When the liver is condemned due to findings not included in any other existing code. For example: telangiectasia, perihepatitis, cirrhosis, stasis. Perihepatitis is registered 77/78 when pleurisy is present at the same time. Fatty liver is registered with code 37/38.
89/90	Mastitis	Mastitis in all animal species. Diagnosis based on visual inspection followed by the incision of mammary lymph nodes.
91/92	Endocarditis	Active, non-encapsulated and chronic endocarditis.

Table S4. Full variance partitioning for most frequent lesions recorded in 2012-2018 at meat inspection of young bulls, sorted by frequency of occurrence. VPC = Variance partition coefficient. Variance levels: A = abattoir, F = farm, B = batch, ε = residual.

Lesion	Prevalence at individual carcass level (%)	VPC_A (%)	VPC_F (%)	VPC_B (%)	VPC_ε (%)
Other pneumonia	5.46	4.67	12.84	11.44	71.05
Pleurisy/pericarditis	5.06	8.21	8.39	8.81	74.59
Common liver fluke	4.3	0.04	71.63	11.56	16.77
Chronic injury	3.4	16.32	9.13	9.15	65.4
Other liver damage	3.2	8.59	3.23	12.59	75.59
Liver abscesses	3.03	0.01	16.81	10.29	72.89
Parasitic liver damage	2.55	15.19	9.58	19.99	55.24
Lancet liver fluke	1.6	0.02	37.45	30.70	31.83
Joint injury	0.71	10.65	10.10	11.37	67.88
Abscess	0.68	0.00	0.00	89.90	10.1
Traumatic peritonitis	0.64	3.78	6.15	15.70	74.37

Table S5. Full variance partitioning for most frequent lesions recorded in 2012-2018 at meat inspection of finishing pigs, sorted by frequency of occurrence. VPC = Variance partition coefficient. Variance levels: A = abattoir, F = farm, B = batch, ε = residual.

Lesion	Prevalence at individual carcass level (%)	VPC_A (%)	VPC_F (%)	VPC_B (%)	$VPC_ε$ (%)
Pleurisy/pericarditis	13.58	0.95	19.49	17.91	61.65
Parasitic liver damage	4.92	1.34	31.03	21.68	45.95
Mycoplasma-like lesions	3.28	6.39	17.74	24.25	51.62
Tail damage	2.71	3.97	7.61	12.57	75.85
Abscess (other than liver)	1.39	1.75	2.21	4.72	91.32
Other cause	1.1	20.48	8.37	11.80	59.35
Other pneumonia	1.04	4.95	5.05	22.03	67.97
Pleurisy and perihepatitis	0.86	2.24	8.37	15.69	73.7
Other liver damage	0.82	3.33	7.30	17.08	72.29
Joint injury	0.74	6.17	7.31	8.77	77.75
Chronic injury	0.72	8.40	3.26	7.92	80.42
Fibrinous pneumonia	0.69	0.00	0.00	69.82	30.18
Acute injury	0.58	17.93	1.62	10.88	69.57
